# Gastrointestinal Delivery of an mRNA Vaccine Using Immunostimulatory Polymeric Nanoparticles

**DOI:** 10.1208/s12248-023-00844-z

**Published:** 2023-08-17

**Authors:** Hyunjoon Kim, Ameya R. Kirtane, Na Yoon Kim, Netra Unni Rajesh, Chaoyang Tang, Keiko Ishida, Alison M. Hayward, Robert Langer, Giovanni Traverso

**Affiliations:** 1 David H. Koch Institute for Integrative Cancer Research, Massachusetts Institute of Technology, Cambridge, Massachusettes 02139, USA; 2 Department of Pharmaceutical Chemistry, University of Kansas, Lawrence, Kansas 66047, USA; 3 Division of Gastroenterology, Brigham and Women’s Hospital, Harvard Medical School, Boston, MA 02115, USA; 4 Department of Bioengineering, Stanford University, Stanford, California 94305, USA; 5 Division of Comparative Medicine, Massachusetts Institute of Technology, Cambridge, Massachusettes 02139, USA; 6 Department of Chemical Engineering, Massachusetts Institute of Technology, Cambridge, Massachusettes 02139, USA; 7 Department of Mechanical Engineering, Massachusetts Institute of Technology, Cambridge, Massachusettes 02139, USA

**Keywords:** GI delivery, mRNA vaccine, nanomedicine, oral vaccine, PBAE

## Abstract

mRNA vaccines can be translated into protein antigens, *in vivo*, to effectively induce humoral and cellular immunity against these proteins. While current mRNA vaccines have generated potent immune responses, the need for ultracold storage conditions (− 80 °C) and healthcare professionals to administer the vaccine through the parenteral route has somewhat limited their distribution in rural areas and developing countries. Overcoming these challenges stands to transform future deployment of mRNA vaccines. In this study, we developed an mRNA vaccine that can trigger a systemic immune response through administration via the gastrointestinal (GI) tract and is stable at 4 °C. A library of cationic branched poly(β-amino ester) (PBAE) polymers was synthesized and characterized, from which a polymer with high intracellular mRNA delivery efficiency and immune stimulation capacity was down-selected. mRNA vaccines made with the lead polymer-elicited cellular and humoral immunity in mice. Furthermore, lyophilization conditions of the formulation were optimized to enable storage under refrigeration. Our results suggest that PBAE nanoparticles are potent mRNA delivery platforms that can elicit B cell and T cell activation, including antigen-specific cellular and humoral responses. This system can serve as an easily administrable, potent oral mRNA vaccine.

## Introduction

Conventional vaccine administration routes, such as subcutaneous (SC) and intramuscular (IM) injection, deliver antigen and adjuvant to antigen-presenting cells (APCs) to elicit humoral and cellular immune responses that kill foreign pathogens (1, 2). Injection-based administration ensures effective delivery across biological barriers; however, needle-based administration can deter indviduals who are afraid of needle sticks and can contribute significantly to the generation of biohazardous sharps. Further, novel vaccine technologies (e.g., mRNA vaccines) need cold storage (< − 20 °C), which can limit access to individuals living in developing countries and rural areas (3–5).

Oral vaccines, administered in the form of tablets, capsules, suspensions, and liquids, are a convenient alternative to systemic injections as they afford vaccine administration in a minimally invasive manner. The gastrointestinal (GI) tract contains immune cell-rich foci, such as Peyer’s patches, and is connected to the draining mesenteric lymph nodes. Both these structures contain immune cells including dendritic cells (DC), macrophages, B cells, and T cells, which are important for the production of immediate and long-lasting immunity (6, 7). Given the convenience and effectiveness of oral vaccines, there are several commercially available oral vaccines including polio, rotavirus, vibrio cholera, and oral typhoid vaccines (8–10). These vaccines generate systemic (IgG) as well as mucosal immunity (IgA), with the latter being critical to target GI infections. This suggests that oral vaccines can be a versatile and potent strategy for certain diseases. Hence, oral vaccines continue to serve as an important tool in vaccination campaigns.

One of the major obstacles in oral vaccine delivery is the degradative, low pH environment in the GI tract. Since most vaccines are protein/peptide biologics, vaccine components can be degraded by digestive enzymes and acids in the stomach (11–13). This can reduce bioavailability of the vaccine and its ability to elicit an immune response. To overcome these challenges, chemical platforms to enhance mucosal permeability (14, 15), devices that penetrate the mucosa (16, 17), and particulate delivery techniques that protect the vaccine from the harsh gut environment (18–22) have been reported. These technologies can be remarkably versatile and could be applied to a range of vaccine platforms.

The antigenic protein can be delivered in various formats including peptides, proteins, viruses, exosomes, and nucleic acids (e.g., mRNA, DNA). Among these, mRNA vaccines are particularly interesting for several reasons (23, 24). First, a small dose of mRNA can generate high amounts of antigenic proteins and trigger antigen specific immunity. Antigenic proteins with diverse physical properties and complex post-translational modifications can be produced (25). Both the mRNA and its delivery excipients can have immunostimulatory activities (26, 27), thus minimizing the need for toxic adjuvants. Additionally, by modifying the mRNA sequence, vaccines for diverse malignancies, including cancer, infectious diseases, and genetic diseases, can be rapidly produced.

Previously, our group has developed an orally ingestible gastric injection device, loaded with polymeric nanoparticles. We showed that this combination can deliver mRNA and transfect gastric cells in a porcine model (28). We build on this finding and investigate if mRNA administration to the GI tract can facilitate the production of T cell, B cell, and antibody responses (Figure S1). Given the high density of immune cells in the GI tract, we hypothesized that oral administration of the mRNA vaccine will elicit a potent humoral and cellular immune response. We surveyed branched hybrid poly(β-amino ester) (referred to as bhPBAE) polymers to determine the optimal nanoparticle formulation with high intracellular mRNA delivery. We formulated nanoparticles by complexing bhPBAE polymers with mRNA and measured transfection efficiency using a high-throughput, flow cytometry assay. First, we investigated the transfection efficiency of bhPBAE nanoparticles in a human colon cancer cell line, Caco-2 (29). We selected polymers with high transfection efficiency and tested their immunostimulatory potency in mouse bone marrow-derived dendritic cells (BMDCs), *in vitro*. These studies facilitated identification of a lead polymer candidate that had the capacity to deliver mRNA and activate innate immune cells. We then showed that lyophilized nanoparticles, made with the lead polymer, could be stored for 2 weeks at 4 °C with minimal loss of activity. Lastly, animal studies showed that gastric as well as small intestinal (SI) administration of the lead nanoparticle formulation yielded antigen-specific immune responses. The magnitude of the immune response was stronger following SI administration. Thus, we have developed a polymeric mRNA vaccine that can be administered into the GI tract, capable of eliciting an antigen-specific B and T cell response. These studies show the feasibility of oral mRNA vaccines to complement current vaccine strategies.

## Materials and Methods

### Cells and Animals

Immunocompetent C57bl/6 mice (female, 6–8 weeks old) were purchased from Charles River Laboratories (Wilmington, USA). Animals were housed in a specific pathogen-free (SPF) facility at MIT which is accredited by American Association for Laboratory Animal Care (AALAC). All animal protocols were approved by the MIT Committee on Animal Care (CAC). A human colon cancer cell line, Caco-2, was purchased from ATCC and regularly tested for mycoplasma contamination. Cells were maintained in DMEM media supplemented with 10% fetal bovine serum (FBS) and 1% antibiotics (100 μg/ml streptomycin, 100 U/ml penicillin). Murine BMDCs were harvested following previously reported protocols (30, 31). Briefly, bone marrow cells were collected from femurs and tibias from C57bl/6 mice and incubated for 6 days with RPMI media supplemented with 10% FBS, 1% antibiotics (100 μg/ml streptomycin, 100 U/ml penicillin), 40 ng/ml GM-CSF (Peprotech), and 20 ng/ml 2-mercaptoenthanol (Sigma) and harvested on day 7 for *in vitro* experiments. Cell culture regents were purchased from Thermo Fisher (Waltham, USA), unless otherwise stated.

### Polymer Synthesis

Linear PBAE polymers and branched hybrid PBAE (bhPBAE) were synthesized as previously reported (28). bhPBAE #844 was prepared as follows: First, two linear PBAE polymers were synthesized by adding an amine and a diacrylate in a glass vial, and stirring the mixture on a hotplate at 90 °C. The two linear polymers used in polymer #844 contained the following combination of amine and diacrylate: linear polymer A (5-amino-1-pentanol + 1,4-butanediol diacrylate) and linear polymer B (3-amino-1-propanol + 1,6-hexanediol diacrylate). Following overnight polymerization, the two linear polymers were dissolved in DMSO at a concentration of 166 mg/ml. Polymers A and B were mixed at 1:1.66 weight ratio. A DMSO-based solution of a branching agent, tris(2-amino ethylamine), was added to the polymer mixture, and samples were placed on a shaker for 24 h at room temperature. Finally, an excess of 1-(3-aminopropyl)-4-methylpiperazine was added as an encapping agent. Characterization of the extent of branching was performed by measuring amount of unreacted branching agent in the reaction mixture as described previously (28).

### Nanoparticle Characterization

Two mRNAs encoding enhanced green fluorescence protein (eGFP) (Cat No. L-7201) and ovalbumin (Cat No. L-7210), respectively, were purchased from Trilink (San Diego, USA). To prepare the nanoparticles (NPs), the stock mRNA solution (as provided by the manufacturer) was diluted in 25 mM sodium acetate buffer (pH 4.2). bhPBAE polymer, dissolved in dimethyl sulfoxide at 100 mg/ml, was further diluted in 25 mM sodium acetate buffer (pH 4.2) to a concetration that was 100 times the concentration of the aqueous mRNA solution. Equal volumes of the polymer and mRNA solution were mixed to achieve a polymer-to-mRNA weight ratio of 100:1. The combination was mixed by rigorous pipetting followed by 15-min incubation at 4 °C.

To measure size and zeta potential, nanoparticles were dispersed at concentration of ~ 10–100 μg/ml and analyzed using dynamic light scatter (Zetasizer, Malvern analytics). To test cytotoxicity, Caco-2 cells were incubated with the nanoparticles overnight. Cell viability was measured using a lactate dehydrogenase (LDH) assay (Thermo Fisher), conducted using the manufacturer’s protocol.

For electron microscopy, 10 μl of the sample and buffer containing solution was dropped on a 200 mesh copper grid (Electron Microscopy Sciences, USA) coated with a continuous carbon film and dried at room temperature. The grid was mounted on a JEOL single tilt holder in the TEM column. Imaging, on the JEOL 2100 FEG microscope, was performed using the largest area on a parallel illumination beam and 10-μm-diameter condenser aperture. The microscope was operated at 200 kV and with a magnification in the ranges of 3000 to 600,000 for assessing particle shape and size and atomic arrangement. All images were recorded on a Gatan CCD camera.

The gel electrophoresis assay was performed based on a previous study (32). Briefly, nanoparticles were prepared with different ratios of eGFP mRNA/polymer (1:50, 1:100). mRNA (1 μg) in DI water, sodium acetate buffer, and nanoparticles were mixed with RNA Gel Loading Dye (ThermoFisher) and boiled at 70 °C for 10 min and cooled, on ice, for 3 min. Samples were then loaded onto a 2% agarose gel in 1 × TBE buffer (ThermoFisher) and run for 90 min at 110 V. Gels were then visualized using a UV transilluminator.

### Caco-2 Transfection

Cells were seeded at a density of 20,000 cells per well in a 96-well plate, and allowed to adhere overnight. Nanoparticles were prepared as described above with eGFP encoding mRNA, and then diluted with serum-free DMEM media. Cells were incubated with the nanoparticles at 100 μg/ml (equivalent to 1 μg/ml of RNA) for 4 h. After the 4-h incubation, nanoparticles were removed and fresh complete RPMI media was added to the cells. Following overnight incubation, cells were detached from the plate and expression of eGFP was measured by flow cytometry (BD Fortessa). A mixture of mRNA and lipofectamine 2000 (ThermoFisher) was used as a positive control.

### BMDC Assays

BMDCs were harvested as described above. Ten thousand cells were seeded in each well of a 96-well plate. First, BMDC transfection was studied using as assay protocol identical to that described for Caco-2 cells. In a separate assay, nanoparticle-mediated immune stimulation was measured. Here, nanoparticles were prepared with ovalbumin encoding mRNA. Nanoparticles were incubated with the BMDCs for 48 h. BMDCs were collected, rinsed, and stained with fluorophore-labeled antibodies (Biolegend, USA). Specifically, CD11c + BMDCs were gated and surface expression of co-stimulatory molecules (CD40, CD80, CD86) and antigen presentation (SIINFEKL:MHCi) were measured using flow cytometry. A schematic of flow cytometry gating is described in Figure S2. Single-cell flow scatter plots of all treatment groups are included in Fig. 3b. The following antibodies were used for flow cytometry: CD11c-APC-Cy7 (clone N418), CD40-FITC (clone 3/23), CD80-BV421 (clone 16–10A1), CD86-PE (clone GL-1), and SIINFEKL:MHCi-APC (clone 25-D1.16).

### Lyophilization

Nanoparticles were prepared with bhPBAE polymer #844 and eGFP mRNA. Sucrose solutions were prepared at concentrations ranging from 5 to 60 mg/ml in 25 mM sodium acetate buffer and mixed with nanoparticles in different ratios. Nanoparticles in sucrose solutions were frozen at − 80 °C for 4 h and lyophilized for ~ 18 h using a bench top lyophilizer (Labconco) with a collector temperature of − 50 °C. The transfection efficiency of lyophilized nanoparticles was characterized in Caco2 cells using the assay described above.

### *In Vivo* Dosing

Ovalbumin mRNA-loaded nanoparticles were prepared using bhPBAE polymer #844 and lyophilized. Lyophilized nanoparticles were dispersed in phosphate-buffered saline (PBS; pH 7.4) before administration. Mice were maintained under anesthesia with isoflurane in oxygen. The ventral abdomen was prepared aseptically for a midline laparotomy. A small section of the small intestine was carefully isolated, and nanoparticles were injected into the small intestinal lumen (20 mg/kg mRNA). All mice were given one pre-operative dose of sustained release buprenorphine subcutaneously and repeated if necessary, 36–48 h later. Seven days after treatment, animals were euthanized, and blood and spleen (SP) samples were collected from each animal. Blood samples were centrifuged; serum was isolated and stored at − 80 °C. Spleen samples were processed for flow cytometry analysis. For intragastric administration of nanoparticles, animals were dosed using an oral gavage needle (20G, 30 mm, Fine Science Tool) without anesthesia. All other experimental procedures were identical to the laparotomy injection study.

### Flow Cytometry

A single-cell suspension of splenocytes was prepared to examine cellular immune response. Splenocytes were prepared and stained with fluorophore-labeled antibodies, as described previously (31). Antibodies used for T cell characterization include CD3-FITC (clone 17A2), CD4-BV510 (clone GK1.5), CD8-APC-Cy7 (clone 53–6.7), CD69-BV421 (clone H1.2F3), CD11a-PE (clone M17/4), and ovalbumin 254–267 tetramer-APC. Antibodies used for B cell characterization include CD19-APC-Cy7 (clone 6D5), CD22-APC (clone OX-97), CD69-BV421 (clone H1.2F3), and MHCii-PE-Cy7 (clone M5/114.15.2). Anti-CD45-BV711 (clone 30-F11) was used to gate immune cells. A flow cytometry gating strategy for B cells and T cells is described in Figure S3. Antibodies were purchased from Biolegend (San Diego, USA), and the tetramer was purchased from MBL International (Woburn, USA).

### ELISA

Serum IgG response against the ovalbumin protein was examined using ELISA. Ovalbumin (Sigma) was dissolved in coating buffer at 10 μg/ml (Biolegend) and coated on a 96-well plate overnight. The plate was then washed with PBS with 0.2% Tween 20 (PBS-T) and blocked with PBS-T with 10% bovine serum albumin for 1 h. Serum samples were diluted with PBS-T and added to the plates for 2 h. The plate was washed to remove unbound serum components. A secondary antibody (anti-IgG-HRP, Thermo) was added to the plate and incubated for 1 h. The unbound secondary antibody was removed, and the HRP substrate solution (Biolegend) was added to the plate. After a 20-min incubation, absorbance was measured at 570 nm using a plate reader (Tecan, USA).

To measure antigen-specific cytokine response, an ex vivo splenocyte assay was performed. Splenocytes were collected and prepared as a single-cell suspension from animals dosed using oral gavage needles. Cells were seeded at 2.5 × 10^6^/ml in a 24-well plate. Either PBS or SIINFEKL peptide (10 μg/ml, invivogen) were added to the wells and incubated for 48 h. Cell supernatant was collected, and IFN-g was measured using ELISA (Biolegend).

### Statistical Analyses and Graphics

One-way analysis of variance (ANOVA) with a post hoc Tukey test (3 + experimental groups) and Student’s *t* test (2 experimental groups) was conducted for statistical analysis using Prism GraphPad software (La Jolla, USA). Results are reported as mean ± standard deviation (SD) or mean ± standard error of mean (SEM). Figure graphics were generated using Biorender software and Microsoft Power-point. *p* value < 0.05 was considered statistically significant. *p < 0.05, **p < 0.01, ***p < 0.001, not significant = n.s. is used for figure presentation.

## Results

### Fabrication and Characterization of PBAE-mRNA Nanoparticles

**Fig. 1:**
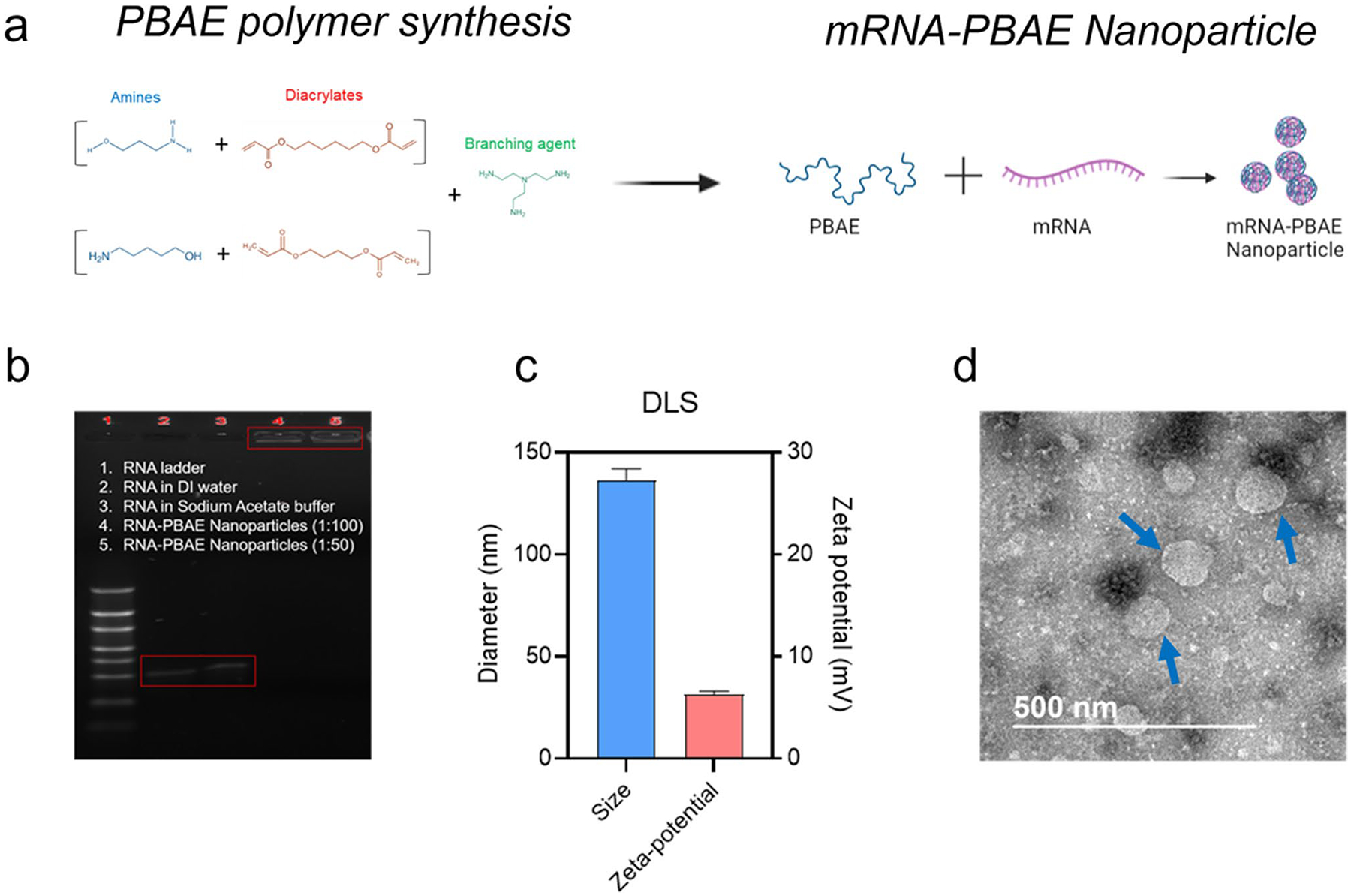
Nanoparticle fabrication using PBAE polymer and mRNA. **a** Schematic of PBAE polymer synthesis and nanoparticle fabrication. **b** A Representative image of gel electrophoresis assay: Column 1: RNA ladder, columns 2 and 3: mRNA, and columns 4 and 5: mRNA-PBAE nanoparticles. **c** Size (diameter, nm) and zeta potential (mV) measured using DLS device. Data is reported as mean ± SD, *n* = 4. **d** A representative TEM image of nanoparticles pointed with blue arrows, scale Bar = 500 nm. **a** Was created with BioRender.com

Cationic bhPBAE polymers can form nanoparticles with negatively charged mRNA as described in Fig. 1a. We examined the transfection efficiency of our nanoparticle library in human colon cancer cells (Caco-2 cells). Similar to our previous reports (28), we observed high eGFP expression in Caco-2 cells with select polymers (Figure S4).

To examine physicochemical properties of the nanoparticles, we selected a nanoparticle formulation fabricated with polymer #844, which demonstrated a high GFP transfection efficiency in the Caco-2 cell screening assay. We performed gel electrophoresis to determine whether mRNA was bound to the bhPBAE polymer (Fig. 1b). While naked mRNA prepared in DI water and sodium acetate buffer were detected at the original molecular weight (232 kDa), mRNA-bhPBAE nanoparticles did not show any signal at 232 kDa. Most of the signal from the nanoparticles was observed in the sampling well, suggesting that the mRNA was complexed with the polymer. Nanoparticles were spherical and had an average hydrodynamic diameter of 136 nm (Fig. 1c, d) and a surface charge of 6.3 mV as determined by both dynamic light scattering (DLS) analysis and transmission electron microscopy (TEM). Toxicity of nanoparticles was examined by measuring cell viability following a 24-h incubation with Caco-2 cells and mouse BMDCs. Nanoparticles showed negligible toxicity, and cell viability was higher than 90% at a nanoparticle concentration of 50 μg/ml (Figure S5).

### PBAE-Based mRNA Delivery to BMDCs

**Fig. 2:**
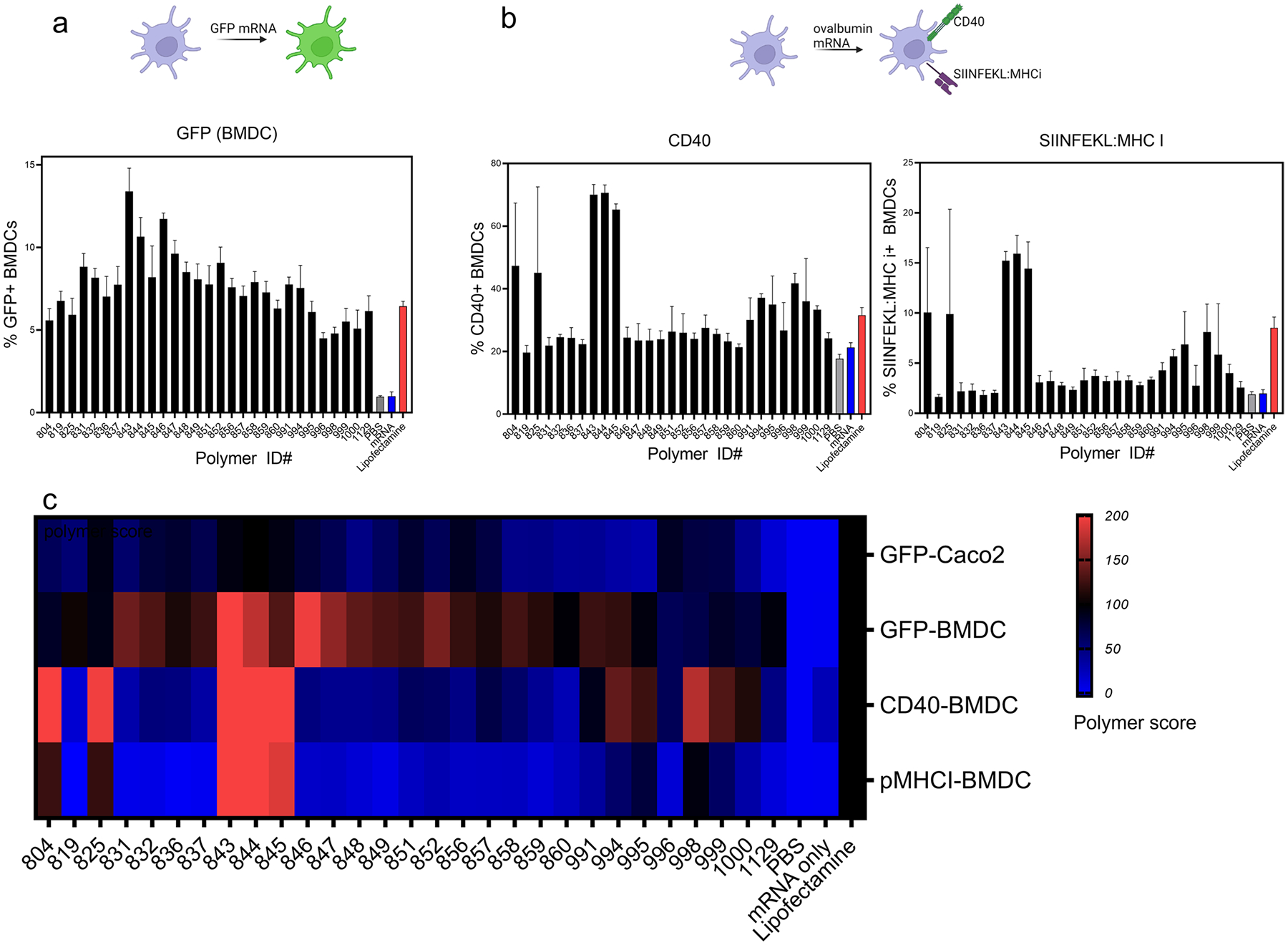
Cellular mRNA delivery using PBAE nanoparticles: **a** Mouse BMDCs were incubated with mRNA (GFP)-PBAE nanoparticles, and GFP expression was measured using flow cytometry. Data is reported as Mean ± SD, *n* = 4. **b** Mouse BMDCs were incubated with mRNA (Ovalbumin)-PBAE nanoparticles, and CD40 and SIINFEKL:MHCi were measured using flow cytometry. data is reported as mean ± SD, *n* = 4. **c** Combined *In Vitro* assays, GFP mRNA delivery in Caco-2 and BMDC and ovalbumin mRNA delivery in BMDC is summarized and presented as a heatmap. Polymer score denotes relative efficiency compared to lipofectamine, which 100 is equal efficiency as lipofectamine. **a** and **b** were created with BioRender.com

We selected 29 polymers that demonstrated high Caco-2 cell transfection efficiency and investigated their transfection and immunostimulation capacity in mouse bone marrow-derived dendritic cells (BMDCs). eGFP mRNA transfection trends observed in BMDCs were similar to those in Caco-2 cells, although the overall % of cells transfected in BMDCs was lower than that in Caco-2 cells (Fig. 2a). GFP + cell frequency in PBAE-treated cells was similar to that of the positive control group—lipofectamine (red column). Particularly, polymers 843, 844, and 846 showed GFP + frequencies of 13.4%, 10.7%, and 11.7%, respectively, which are ~ twofold higher than lipofectamine.

We then tested cellular delivery of mRNA encoding a model antigen, ovalbumin (MW 375 kDa). In this assay, we measured expression of the co-stimulatory molecule, CD40, and SIINFEKL:MHCi using flow cytometry (Fig. 2b). Successful delivery of ovalbumin mRNA will produce ovalbumin in the cytosol, which can be processed and displayed on the cell surface MHC receptor. Furthermore, we (33) and others (27, 34) have demonstrated nanoparticle- and polymer-mediated activation of immune cells. This is expected to upregulate the levels of CD40 on the cell surface. Hence, we probed the expression of MHC:SIINFEKL and CD40 on the DC surface. Unlike the GFP transfection assay, where PBAE polymers and lipofectamine showed comparable efficacy, CD40 expression was much higher with the PBAEs as compared to lipofectamine. Specifically, nano-particulate delivery of ovalbumin mRNA using polymers 843, 844, and 845 induced significantly increased levels of CD40 on BMDCs (70.1%, 70.6%, and 65.3%, respectively), compared to naked mRNA and lipofectamine groups (21% and 31%, respectively). Expression of SIINFEKL:MHCi showed similar trend as CD40, where polymers 843, 844, and 845 facilitated high expression of SIINFEKL:MHCi to 15.2%, 15.9%, and 14.4%, respectively, while naked mRNA and lipofectamine-delivered groups showed 1.9% and 8.5%, respectively.

*In vitro* assay data is described in Fig. 2c. Next, mRNA delivery efficiency of PBAE polymers was scored relative to that of lipofectamine. A score of 100 indicates performance identical to lipofectamine. Among the polymers tested, 19 polymers (819, 831, 832, 836, 837, 847, 848, 849, 851, 852, 856, 857, 858, 859, 991, 995, 998, 999, 1000) showed a polymer score higher than 100, in at least one category, while polymers 804, 825, and 944 had polymer scored higher than 100 in two categories (CD40 and SIINFEKL:MHCi). Polymers 843, 844, and 845 showed polymer scores higher than 100 in three categories (CD40, SIINFEKL:MHCi, BMDC-GFP). Based on the screening result, polymer 844, which is the intermediate form of polymers 843 and 845, was selected for further formulation optimization and *in vivo* animal studies.

### Immunostimulatory Effect of PBAE Polymers

**Fig. 3:**
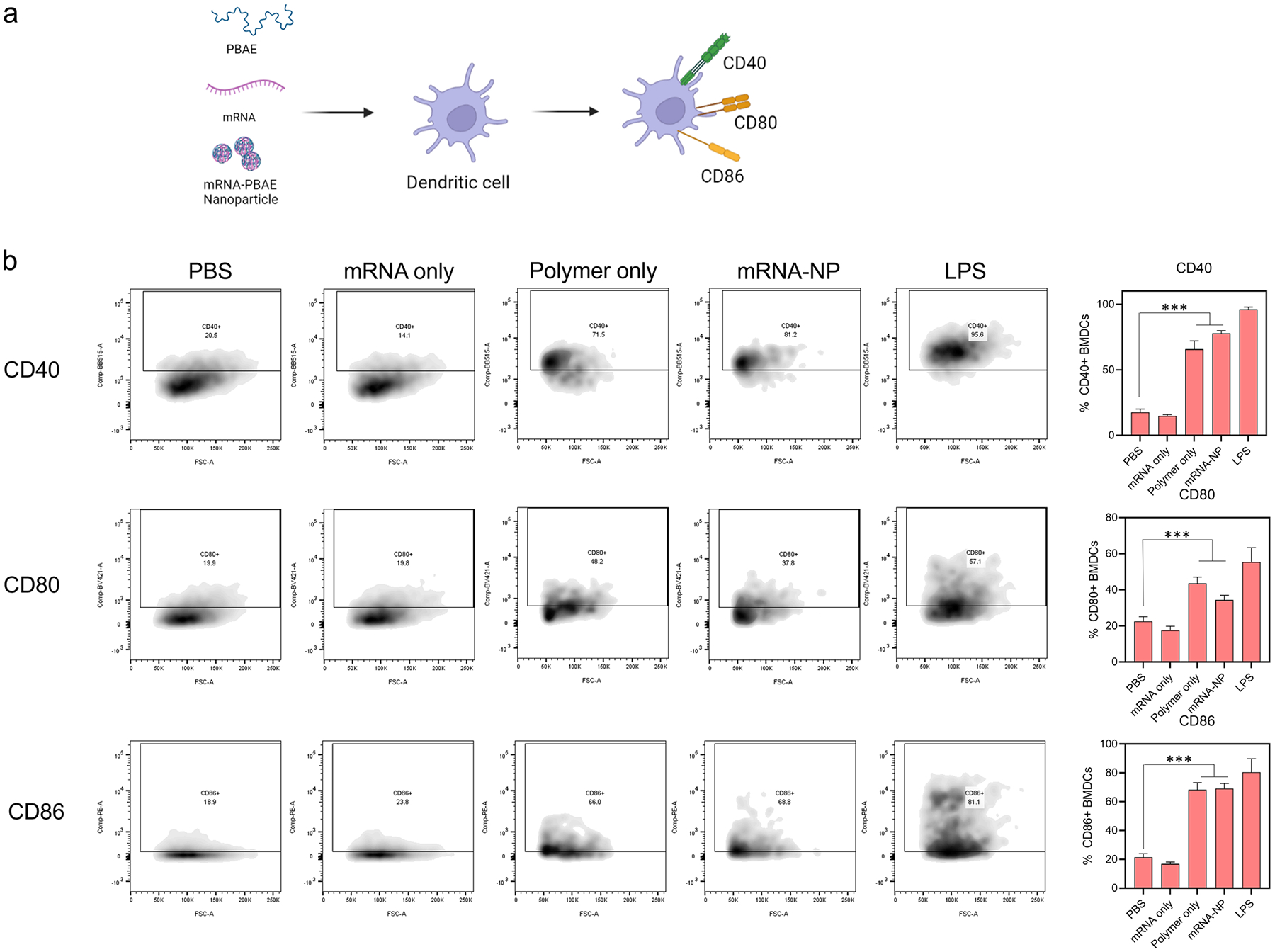
PBAE polymer induces immunostimulatory response. **a** Schematic of BMDC assay to examine effect of mRNA alone, PBAE alone, and mRNA-PBAE nanoparticle is shown. **b** Flow cytometry analysis of co-stimulatory molecules (CD40, CD80, CD86) on BMDC (Left: scattering plot, right: columnar images) is shown. Data is reported as mean ± SD, *n* = 4. **a** was created with BioRender.com

Upregulation of CD40 and MHCi:SIINFEKL by ovalbumin mRNA delivery, using mRNA-PBAE nanoparticles, is indicative of its immunostimulatory capacity. To investigate the origin of these effects, we repeated BMDC activation assays with treatment groups including mRNA only, PBAE only, and mRNA-PBAE nanoparticles. For each of these groups, levels of co-stimulatory molecules CD40, CD80, and CD86 (Fig. 3a) were measured. As shown in Fig. 3b, mRNA alone did not induce upregulation of any co-stimulatory molecules, likely because we used uridine-substituted mRNA. However, PBAE alone induced a threefold increase in CD40 expression, twofold increase in CD80 expression, and threefold increase in CD86 expression, all of which are similar to the mRNA-PBAE nanoparticle-treated groups. This suggests that the immunostimulatory effect likely stems from the polymer itself and underscores the potential of these materials for vaccine development.

### Optimization of Lyophilization to Enhance Long-Term Stability

**Fig. 4:**
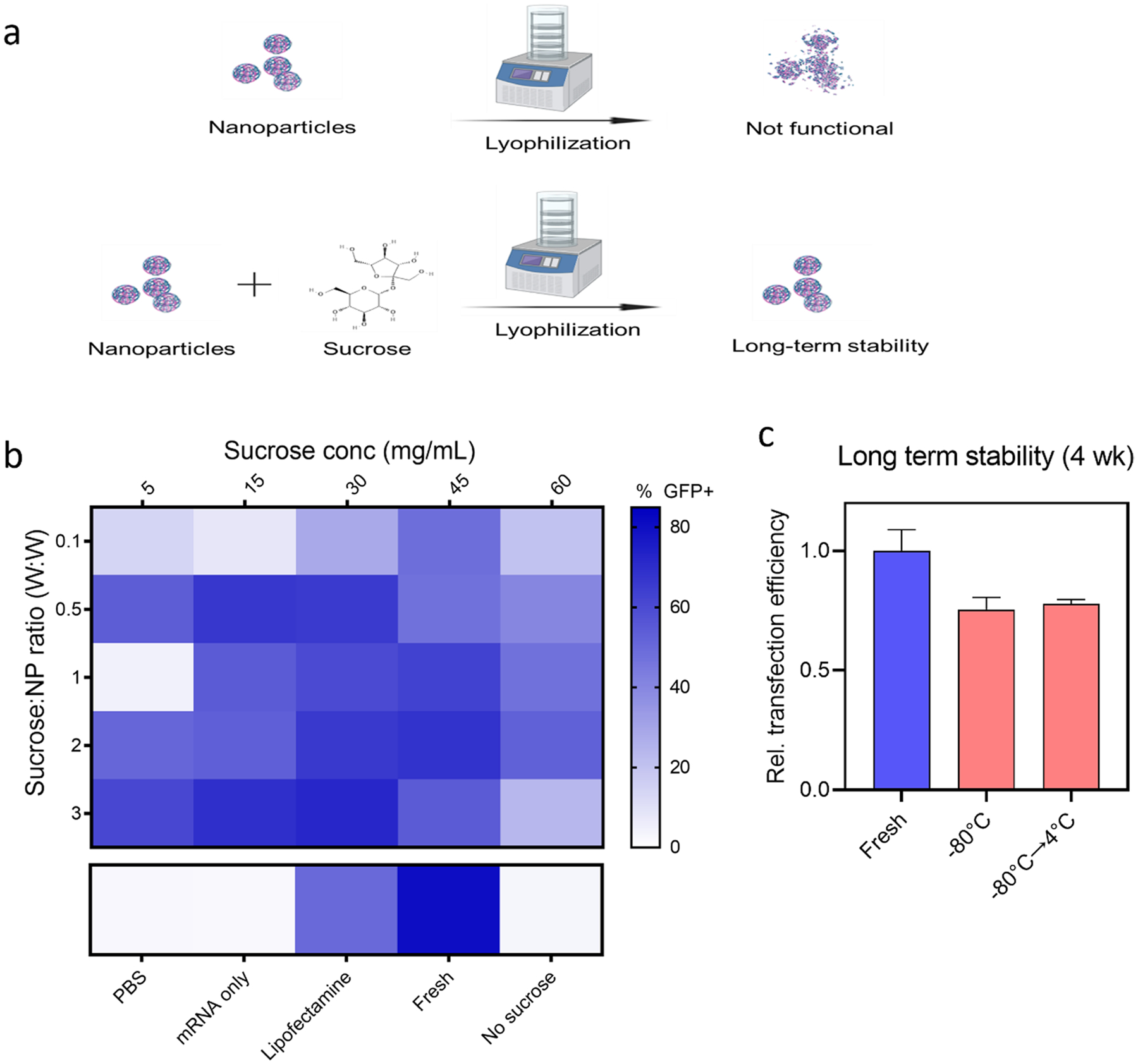
Lyophilizing PBAE nanoparticles with sucrose enables long-term storage. **a** Schematic of lyophilization of nanoparticles with sucrose is shown. **b** Lyophilized nanoparticles were examined for GFP transfection assay using caco-2 cells to validate stability. Heatmap demonstrates % GFP cells measured by flow cytometry, *n* = 4. **c** Nanoparticles lyophilized with sucrose (15 mg/ml, 0.5:1 ratio) and stored at designated temperature were examined for caco-2 transfection (GFP +) 4 weeks after fabrication. Data is reported as mean ± SD, *n* = 4. **a** was created with BioRender.com

For long-term storage of mRNA-bhPBAE nanoparticles, stabilization in a solid state can be advantageous. Sucrose, a disaccharide cryoprotectant, was utilized in this assay. Sucrose solutions of different concentrations were mixed with mRNA(eGFP)-bhPBAE nanoparticles at different volume ratios before lyophilization (Fig. 4a). Lyophilized nanoparticles were added to Caco-2 cells, and GFP transfection efficiencies were examined and compared with fresh nanoparticles (Fig. 4b). As expected, nanoparticles lyophilized without sucrose showed no GFP transfection (3% GFP +), which is similar to the basal GFP signal detected in PBS-treated group (2.5% GFP +). Although no lyophilized formulation outperformed the fresh nanoparticles (80.5% GFP +), 17 formulations showed GFP transfection efficiency greater than lipofectamine treatment (49.8% GFP +), which demonstrates that sucrose is an effective cryoprotectant for this formulation. Among the tested groups, sucrose (15 mg/ml) added to nanoparticles at a 0.5:1 volumetric ratio showed 66.3% GFP + cells, and was selected for further *in vivo* characterization. This condition required lower amounts of total sucrose compared to other high-performing formulations (60 mg/ml 0.5:1 ratio, 60 mg/ml 1:1 ratio, 45 mg/ml 2:1 ratio). We then examined the stability of lyophilized mRNA-bhPBAE nanoparticles over a period of 4 weeks (Fig. 4c). An aliquot of nanoparticles was stored directly at − 80 °C for 4 weeks while a second aliquot was first stored at − 80 °C for 2 weeks, followed by storage at 4 °C for 2 weeks. Lyophilized nanoparticles were tested using the Caco-2 cell transfection assay, and both nanoparticle groups showed 75% relative transfection efficiency compared to fresh nanoparticles.

### B Cell and IgG Responses Following Small Intestine Delivery of Nanoparticles

**Fig. 5:**
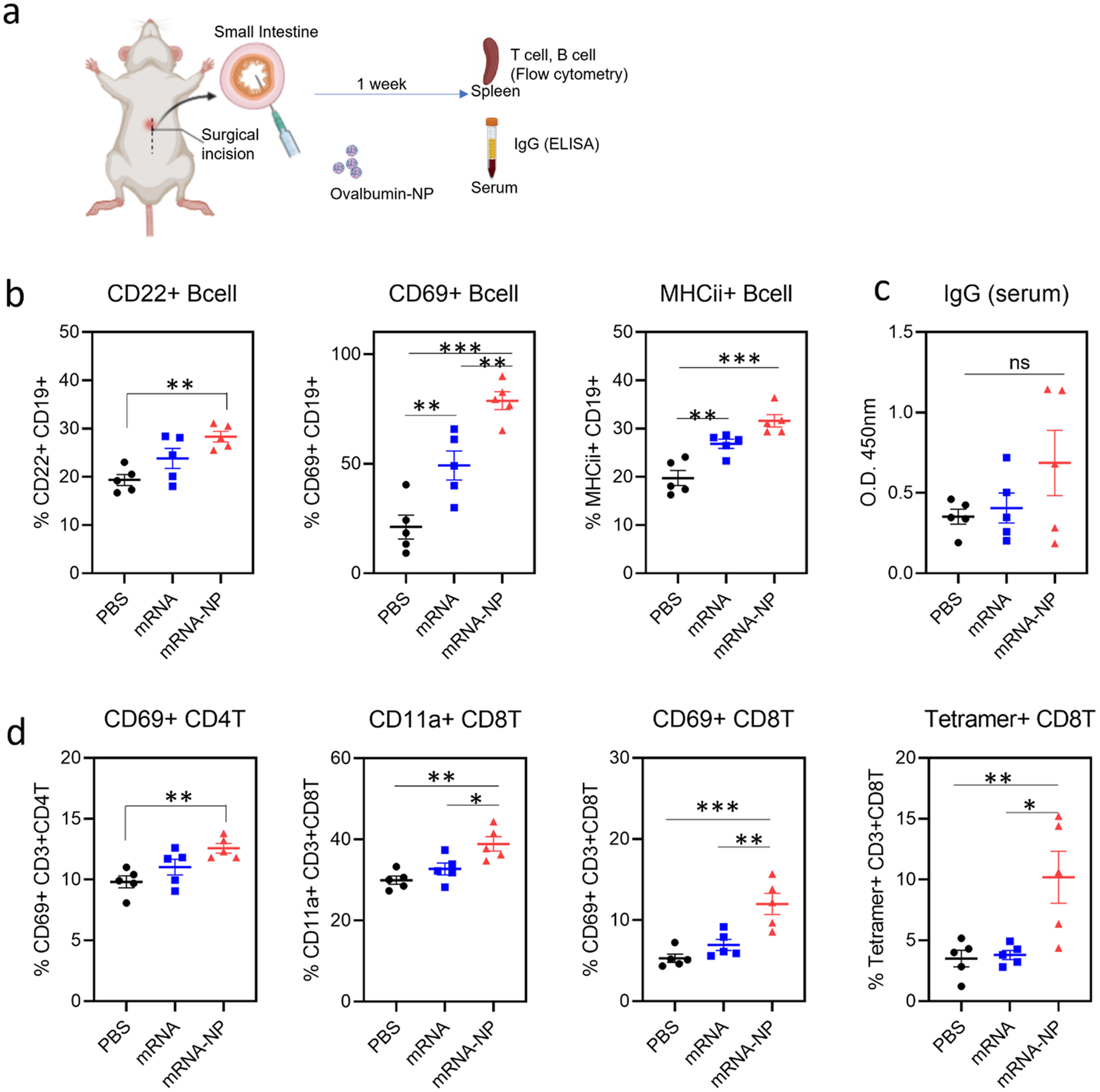
Small intestine delivery of mRNA-PBAE nanoparticle elicits B cell and T cell immunity. **a** schematic of GI administration procedure via laparotomy is shown. **b** Frequency of B Cells expressing CD22, CD69, and MHCii is shown. Data is reported as Mean ± SEM, *n* = 5. **c** ovalbumin-specific IgG was measured by ELISA and reported in absorbance (OD 450 nm). Data is reported as mean ± SEM, *n* = 5. **d** Frequency of CD69 + CD4T cells, CD11a + CD8 T cells, CD69 + CD8 T cells, and SIINFEKL-tetramer + CD8 T cells is shown. Data is reported as mean ± SEM, *n* = 5. **a** was created with BioRender.com

To investigate the immunostimulatory activity of mRNA-bhPBAE nanoparticles, we administered nanoparticles (with ovalbumin mRNA) into the small intestine of immunocompetent mice. We characterized the cellular (B cell, T cell) immune response through splenocyte analysis (flow cytometry) while humoral (IgG) response was measured through serum analysis (ELISA) as described in Fig. 5a.

B cell activation was evaluated using activation markers CD22, CD69, and MHCii (35) (Fig. 5b). Compared to animals treated with PBS and mRNA-only, showing 19.3% and 23.8% of CD22 + B cells, respectively, nanoparticle-treated animals showed 28.3% of CD22 + B cells. Interestingly, mRNA treatment increased frequency of CD69 + B cells to 49.1%, which is twofold higher than PBS (22.1%). Further, nanoparticle treatment showed the highest CD69 + B cell frequency at 78.7%. PBS-treated animals had the lowest frequency of MHCii expression on B cells, at 19.7%, while mRNA- and nanoparticle-treated animals had frequencies of 26.8% and 31.6%, respectively.

We performed ovalbumin-specific serum IgG ELISA to investigate if B cell activation led to an antigen-specific humoral response. As shown in Fig. 5c, serum IgG levels were higher in the mRNA-NP-treated groups (0.69 avg absorbance) compared to mRNA-only (0.4 avg absorbance) and PBS (0.3 avg absorbance) groups, although the differences were not statistically significant.

### CD4 and CD8 T Cell Responses Following Small Intestine Delivery of Nanoparticles

Activation of CD4 helper T cells was measured using flow cytometry (Fig. 5d). We observed that the frequency of CD69 + CD4 T cells was increased in mice that received nanoparticle treatment (12.6%), compared to PBS (9.8%) and mRNA-only (11%) treated mice. CD8 T cell activation, measured by CD11a expression (36), showed a similar trend where PBS- and mRNA-treated animals showed frequencies of 29.9% and 32.7%, respectively. However, frequency of CD11a + CD8 T cells in the mRNA-NP-treated mice was 38.9%, which is significantly higher than other groups. Frequency of CD69 + CD8 T cells was also the highest in animals treated with nanoparticles (12%) compared to the PBS (5.3%) and mRNA-only (6.9%) groups. Upon confirmation of successful CD4 and CD8 T cell activation following nanoparticle treatment, antigen-specific CD8 T cells were measured using SIINFEKL tetramers which can identify CD8 T cells that are primed by the ovalbumin antigen. PBS-treated animals and mRNA-treated animals showed 3.5% and 3.8% tetramer + CD8 T cells, respectively. Nanoparticle-treated animals showed a drastically higher frequency of 10.2% tetramer + CD8 T cells. Our data suggest that nanoparticles are not only able to activate general T cell responses but also facilitate antigen-specific CD8 T cell response.

### Immune Responses Following Intragastric Delivery of Nanoparticles

**Fig. 6:**
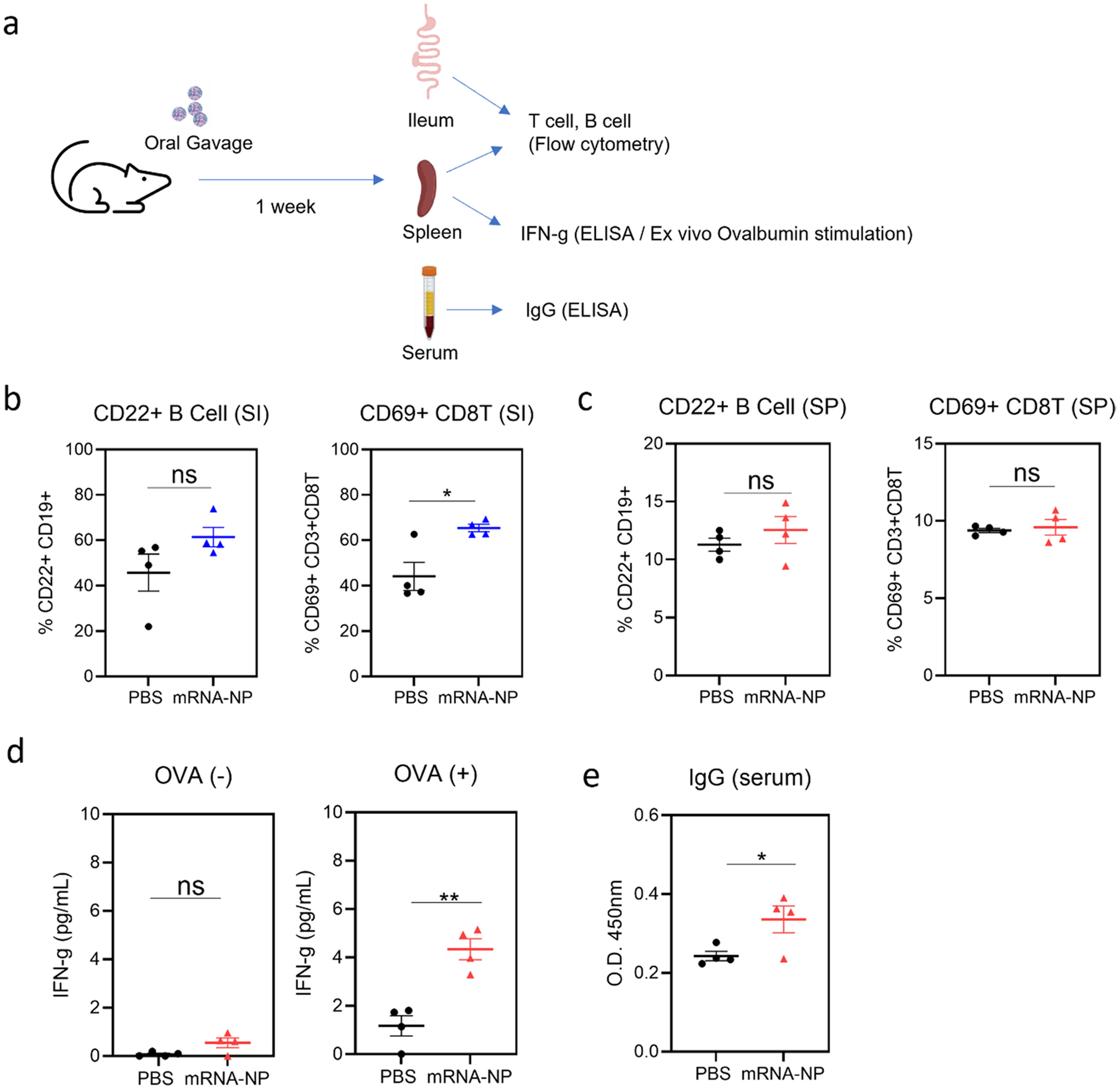
Gastric delivery of mRNA-PBAE nanoparticle triggers antigen-specific immune response. **a** Schematic of experimental design is shown. **b** Frequency of CD22 expressing B cells and CD69 expressing CD8 T cells in the small intestine is shown. Data is reported as mean ± SEM, *n* = 4. **c** Frequency of CD22 expressing B cells and CD69 expressing CD8 T cells in the spleen is shown. Data is reported as mean ± SEM, *n* = 4. **d** Splenocytes were incubated with PBS (OVA −) or SIINFEKL peptide (OVA +), and IFN-g was measured using ELISA. Data is reported as mean ± SEM, *n* = 4. **e** Ovalbumin-specific IgG was measured by ELISA and reported in absorbance (OD 450 nm). Data is reported as mean ± SEM, *n* = 4

As SI delivery of nanoparticles demonstrated enhanced B cell and T cell activation, we performed a separate animal study experiment in which the nanoparticles were delivered into the stomach using oral gavage needles. This is more clinically relevant than a laparotomy injection (Fig. 6a). In this experiment, we measured B cell and T cell responses from the small intestine and spleen. Interestingly, while B cell and T cell activation in the spleen was negligible, we observed enhanced CD69 + CD8 T cell (*p* < 0.05) and a slight increase in CD22 + B cells in the SI (Fig. 6a, b). Ex vivo splenocyte stimulation assays demonstrated that splenocytes from the nanoparticle-treated animals induce antigen (ovalbumin)-specific IFN-g responses (Fig. 6d). Antigen-specific IgG levels in the sera of nanoparticle-treated animals were increased compared to those of PBS-treated animals.

## Discussion

mRNA is a versatile vaccine modality, which serves as a template to produce the antigen of interest, following intracellular delivery. Compared to attenuated and live vaccines, the manufacturing of mRNA vaccines is relatively quick and convenient as a diverse set of antigens can be produced with relatively similar fabrication techniques (37). Additionally, mRNA is a natural adjuvant, capable of activating immune cells via TLR3 (double-stranded RNA), TLR7 (single-stranded RNA), and TLR8 (single-stranded bacterial RNA) (38). While these features support the use of mRNA as a potent vaccine modality, mRNA is easily degraded by RNases and maintaining its stability *in vivo* is challenging (39). In recent years, lipid nanoparticles (LNPs) have been used in the clinic as an mRNA vaccine platform, which has been efficacious in increasing IgG titer (23, 24). Recent efforts to facilitate targeted delivery of RNA demonstrates the potential of mRNA vaccine for diverse malignancies and suggests a need for mRNA platforms that are modified depending on the size of mRNA, target cells/tissues, and disease models (40, 41). As we reported previously, bhPBAE polymers are cationic and can easily form a nanoparticle with nucleic acids including DNA and mRNA (28). In addition, we observed a cell line-dependent trend in polymers that showed the highest transfection efficiency. This suggests that we can fabricate a mRNA platform using PBAE polymers, which is optimized for a given target cells/tissues, and its administration route. Hence, we screened the bhPBAE polymer library to optimize for mRNA delivery in an oral vaccine application.

As shown in Figure S2, we identified bhPBAE polymers that efficiently transfected Caco-2 cells, a platform commonly used to assess transfection capacity in the GI tract (29). We then shortlisted bhPBAE polymers to assess immune cell activation. In general, GI immunity is skewed towards tolerance rather than activation. Particularly, tolerogenic DCs induce regulatory T cell responses, to maintain homeostasis (7). Therefore, we tested if bhPBAE polymers, which were effective in Caco-2 cells, are also capable of entering antigen-presenting cells and inducing activation, using the assays described in Fig. 2. While all polymers were effective in delivering GFP mRNA to BMDCs, only a few bhPBAE polymers were capable of delivery of ovalbumin mRNA. Successful intracellular delivery of ovalbumin mRNA will produce immunogenic ovalbumin protein within BMDCs. This will trigger expression of co-stimulatory molecule CD40, and SIINFEKL:MHC I, an antigen cross-presentation immunocomplex that can initiate antigen-specific CD8 T cell responses. These markers of antigen presentation were elevated in all of the polymers highlighted in Fig. 2b. Combining this data, we selected polymer #844, which demonstrated a high performance score in all *in vitro* assays, for further characterization. Interestingly, when we treated the BMDCs with only polymer #844, we observed increased expression of co-stimulatory molecules CD40, CD80, and CD86. The level of expression seen was similar to that of BMDCs treated with the polymer nanoparticle complexes. This shows that polymer #844 enhances intracellular delivery of mRNA and also facilitates the activation of immune cells. Although we have not elucidated the immunostimulatory mechanism of bhPBAE polymer #844, we presume that it triggers a pattern recognition receptor (PRR), previously reported by other immunostimulatory polymers and polysaccharides in literature (42–44).

At this stage, mRNA and bhPBAE polymers were each prepared in an aqueous phase suspension for *in vitro* characterization, which limits stability of the nanoparticle complexes. To stabilize the mRNA for long-term storage, LNPs can be lyophilized with sucrose as a cryoprotectant. This methodology was employed to optimize the mRNA-bhPBAE nanoparticle formulations’ stability (45, 46). As demonstrated in Fig. 4, we prepared sucrose solutions with varying concentrations, and mixed it with nanoparticles prior to lyophilization. Interestingly, there were certain sucrose concentrations and sucrose-to-nanoparticle ratios which yielded increased nanoparticle stability. In this study, we have identified that 15 mg/ml sucrose solution, added at a 0.5:1 volume ratio, and 60 mg/ml sucrose solution, added at 0.5:1 volume ratio, provided the best nanoparticle stability. This was confirmed by evaluating Caco-2 transfection efficiency of lyophilized samples, compared to freshly prepared nanoparticles. Among the two concentrations, we selected 15 mg/ml solution to reduce the total amount of sucrose in the formulation and further tested the stability of the formulation over the course of 4 weeks. Lyophilized nanoparticles that were stored at − 80 °C for 4 weeks (currently used in the clinic) and nanoparticles stored at − 80 °C for 2 weeks followed by 4 °C for 2 weeks both showed high transfection efficiency. While the result is promising, recent studies report methods to stabilize mRNA vaccines at 4 °C and room temperature storage (47). Hence, further efforts to modify the cryoprotectant (e.g., trehalose), expanding the polymer library to include polymers similar to 844, and hybrid PBAE nanoparticles fabricated with lipids, polysaccharides, and other polymers are required to address these storage challenges.

For *in vivo* study, we began by directly delivering the mRNA nanoparticles into the lumen of the small intestine via a laparotomy (48). This method was employed to eliminate potential degradation in the stomach. In this study, we focused on validating whether mRNA-PBAE nanoparticles could elicit immune responses when delivered directly to the small intestine. Furthermore, delivery to the small intestine can be achieved by loading nanoparticles in conventional enteric coated capsules or using smart capsules (49, 50), a direction that will be assessed in future studies. As demonstrated in Fig. 5, ovalbumin mRNA nanoparticles resulted in significantly higher B cell and T cell marker expression. Additionally, serum ovalbumin IgG levels (not significant) and ovalbumin-specific CD8 T cells (*p* < 0.01 *vs* PBS) were higher in animals immunized with nanoparticles. This data clearly demonstrates that nanoparticles can trigger general B cell and T cell activation; however, antigen-specific responses showed sub-optimal efficacy with high variation between samples. Interestingly, mRNA alone enhanced B cell activation compared to PBS control groups. However, only nanoparticles were effective in T cell activation while mRNA alone had no effect. Since B cells can function as antigen-presenting cells, it is possible that mRNA and mRNA nanoparticles were directly internalized by B cells, thereby inducing strong B cell activation (51, 52). Nonetheless, T cells are mainly activated by antigen-presenting cells (53, 54). Thus, we can presume that only mRNA nanoparticles, but not mRNA alone, successfully delivered mRNA to the DCs and macrophages, leading to downstream T cell stimulation. Although general B cell and T cell activation can be triggered by mRNA delivery and immunostimulatory polymers, antigen-specific responses demonstrate that ovalbumin mRNA successfully produced ovalbumin antigen in the GI tract, only when delivered in bhPBAE nanoparticles.

Our hypothesis was further validated in the gastric injection study, a more clinically relevant oral dosage route compared to a laparotomy injection. Interestingly, we did not observe significant B cell and T cell responses, when compared with the laparotomy injection study. It is possible that the laparotomy procedure causes inflammation in the GI tract and intraperitoneal regions (55, 56), thus increasing the basal levels of activation markers in cellular immune responses. Nonetheless, we observed antigen-specific IgG and IFN-g responses after intragastric injection. Interestingly, while oral gavage treatment of nanoparticles induced a mild but statistically significant IgG response, laparotomy injection of nanoparticles induced a highly variable response where a few animals showed increased immune response and others showed no response. Variability in IgG response of laparotomy-injected nanoparticles can be due to variability during injection, and the data suggests that optimized delivery of nanoparticles in the GI tract can induce a potent IgG response. It should be noted that we have limited the oral gavage to a single dose and sampling at day 7 to be consistent with the laparotomy injection study. This suggests that an optimized dosing schedule and long-term characterization of IgG responses should be investigated in an oral gavage model. Combined *in vivo* studies suggest that mRNA nanoparticles can induce antigen-specific cellular and humoral immunity and can serve as a platform for oral vaccine delivery.

## Conclusion

Application of mRNA vaccines can be further expanded with formulations that target specific cells and tissues and can be delivered using different routes of administration. PBAE nanoparticles demonstrated robust intracellular delivery of mRNA, great potential in eliciting general B cell and T cell activation, and in successful generation of antigen-specific immune responses. Future directions include studies to validate the safety and toxicity of this oral vaccine modality, with specific focus on prophylactic and therapeutic efficacies in an animal disease model (e.g., influenza, GI cancer). These studies can further demonstrate the clinical feasibility of an oral mRNA nanoparticle vaccine.

## Supplementary Material

Supplement

## Data Availability

Data will be available on request.
