# Supplementary material for: Gastrointestinal Delivery of an mRNA Vaccine Using Immunostimulatory Polymeric Nanoparticles
Source: AAPS J. Author manuscript; Available in PMC 2024 Feb 17. (PMC10845796; doi:10.1208/s12248-023-00844-z)
Supplement: Supplement [file NIHMS1962887-supplement-Supplement.docx]

**Gastrointestinal delivery of an mRNA vaccine using immunostimulatory polymeric nanoparticles**

Hyunjoon Kim^1,2^, Ameya R. Kirtane^1,3^, Na Yoon Kim^1^, Netra Unni Rajesh^1,4^, Chaoyang Tang^1^, Keiko Ishida^1,3^, Alison M. Hayward^1,5^, Robert Langer^1,6^, Giovanni Traverso^1,3,7^*

^1^David H. Koch Institute for Integrative Cancer Research, Massachusetts Institute of Technology, Cambridge, MA 02139, USA

^2^Department of Pharmaceutical Chemistry, University of Kansas, Lawrence, KS, 66047, USA

^3^Division of Gastroenterology, Brigham and Women’s Hospital, Harvard Medical School, Boston, MA, 02115, USA

^4^Department of Bioengineering, Stanford University, Stanford, California, 94305, USA

^5^Division of Comparative Medicine, Massachusetts Institute of Technology, Cambridge, MA 02139, USA

^6^Department of Chemical Engineering, Massachusetts Institute of Technology, Cambridge, MA 02139, USA

^7^Department of Mechanical Engineering, Massachusetts Institute of Technology, Cambridge, MA 02139, USA

^*^Corresponding author. E-mail: cgt20@mit.edu (G.T.)

**Keywords** mRNA vaccine, oral vaccine, nanomedicine, GI delivery, PBAE
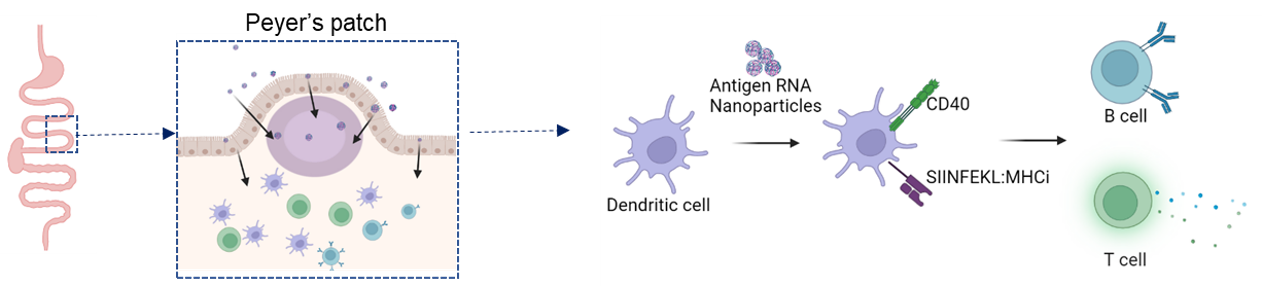
Figure S1. Schematic of B cell and T cell activation following GI-delivery of mRNA-nanoparticle is shown. Figure S1 was created with BioRender.com


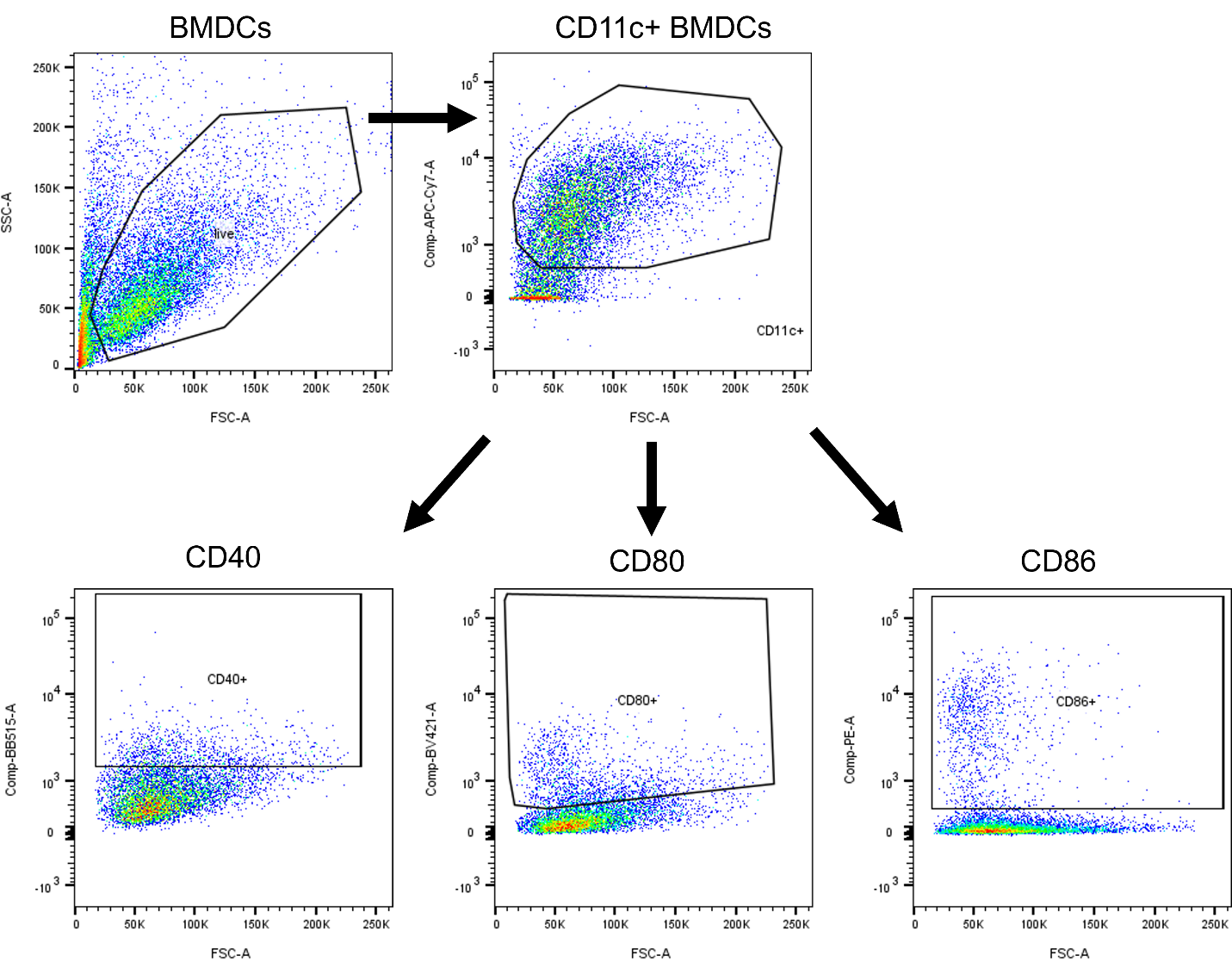


Figure S2. Schematic of flow cytometry gating strategy for BMDC analysis is shown


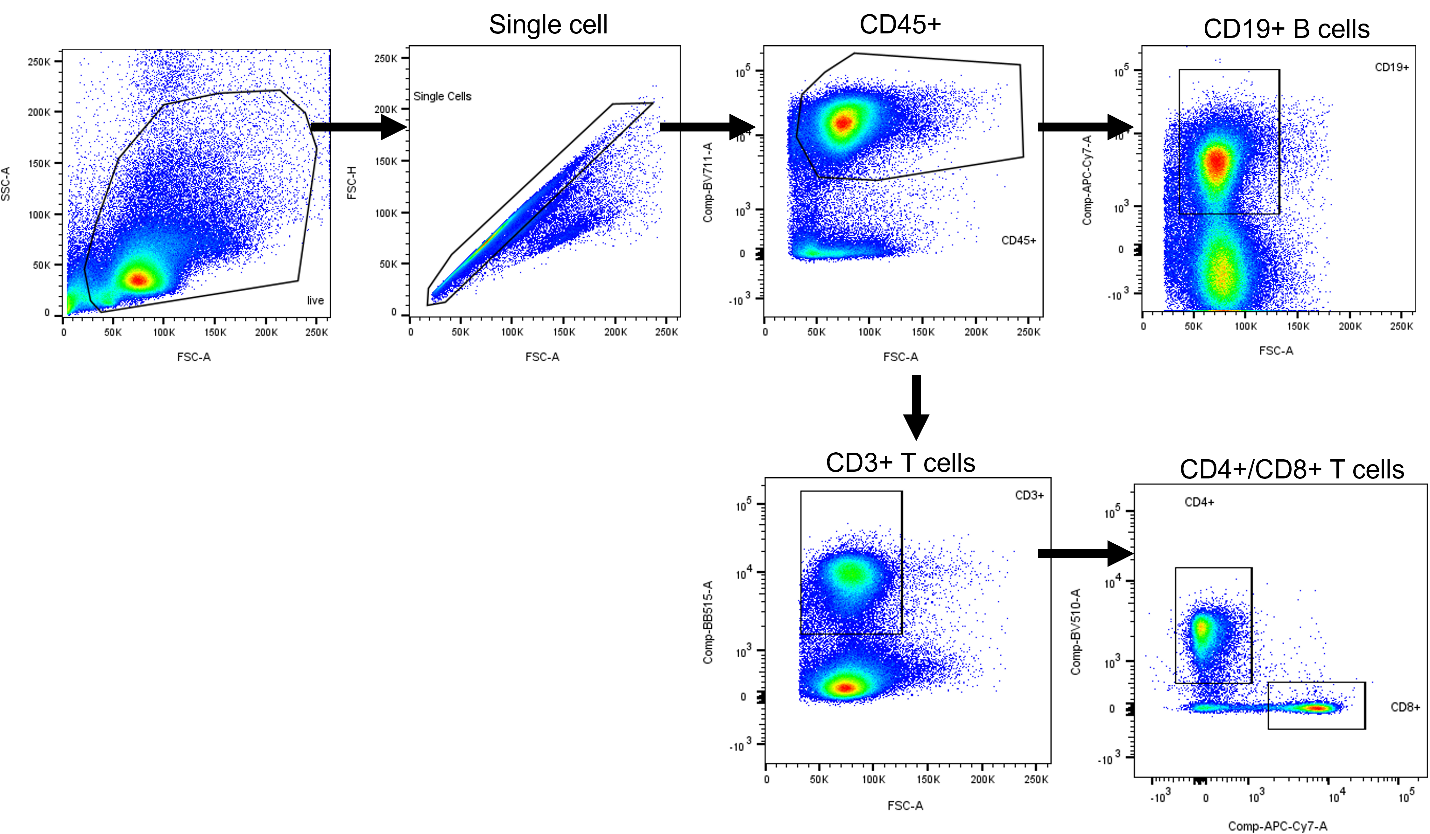


Figure S3. Schematic of flow cytometry gating for in vivo B cell and T cell analysis is shown


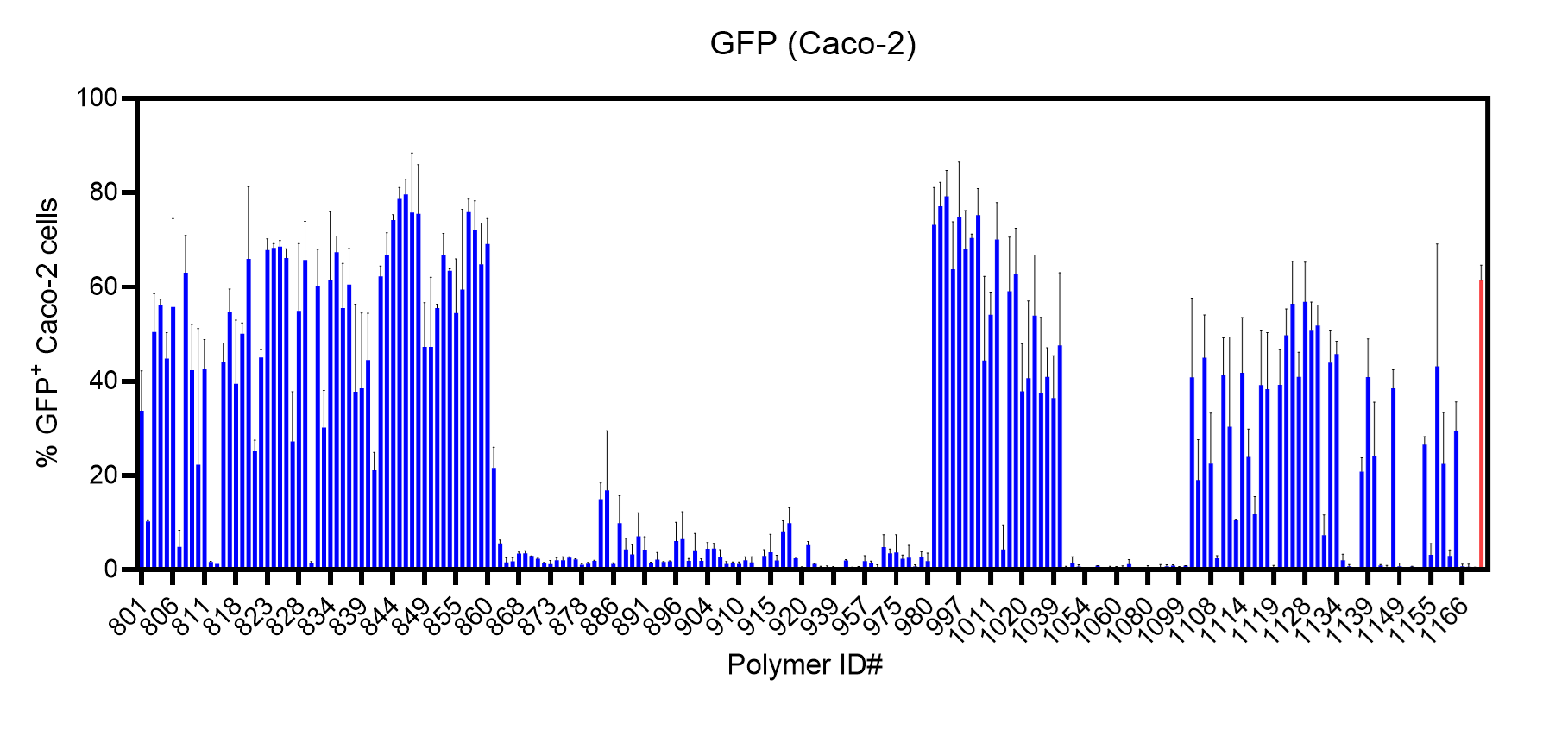


Figure S4. PBAE library was tested for GFP transfection using Caco-2 cells. Data is reported as mean ± SD, n=6. Samples are presented as blue columns and lipofectamine (positive control) is presented as red.


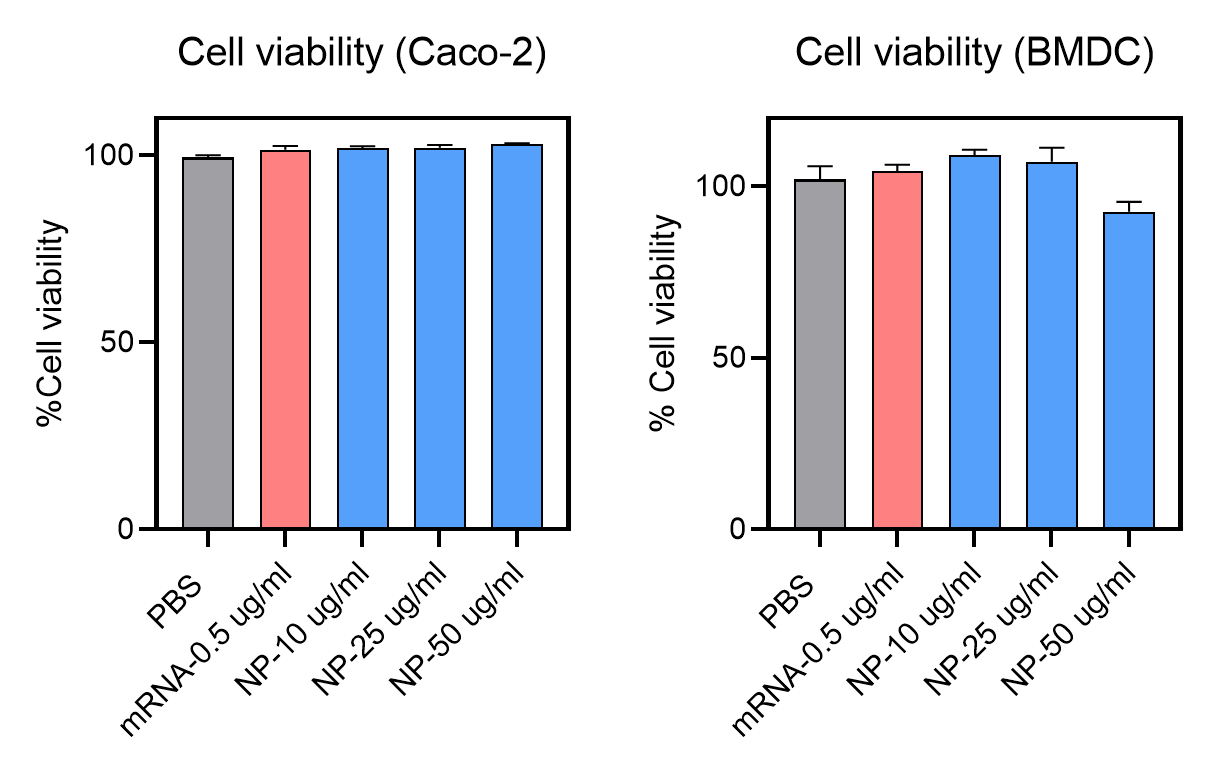


Figure S5. Cell viability was measured using LDH assay to examine toxicity of nanoparticles. Data is reported as mean ± SD, n=4
